# Mobilizing Elders: An Interprofessional Effort

**DOI:** 10.1093/geroni/igab046.3178

**Published:** 2021-12-17

**Authors:** Andrew de la Paz, David Lynch, Carissa Lau, Kimberly Mournighan, John Batsis, Laura Hanson, Maureen Dale, Kimberly Young

**Affiliations:** 1 UNC, Chapel Hill, North Carolina, United States; 2 University of North Carolina, Chapel Hill, North Carolina, United States; 3 University of North Carolina at Chapel Hill, Chapel Hill, North Carolina, United States; 4 University of North Carolina, University of North Carolina, North Carolina, United States

## Abstract

Older adults often experience functional decline during hospitalization as a result of immobility. Such decline has associated adverse outcomes, including gait instability, falls, pressure injuries, delirium, and new nursing home admissions. Our objective was to create an effective and sustainable in-hospital mobility program through enhanced interdisciplinary cooperation in an Acute Care of the Elderly (ACE) unit. An interdisciplinary team at UNC’s 25-bed ACE unit planned and delivered enhanced patient mobility beginning in July 2020. We used an input-process-output model to design and analyze an intervention based on enhanced collaboration. Inputs included a mobility taskforce which was comprised of physicians, nurses, physical and occupational therapists, and quality improvement specialists. Through regular meetings, each taskforce member contributed to the study design and were empowered to identify barriers to implementation. Outputs included stakeholder engagement and mobility rates. Early results show a doubling in mobility rates over a 6-month period with consistent and enthusiastic stakeholder engagement. Observations of such benefits include: a) stakeholder inclusion from each discipline ensured implementation that was pragmatic and easily incorporated into the daily workflow; b) mobility champions regularly disseminated information to their respective disciplines, leading to changes using a quality improvement process; and c) barriers to implementation were rapidly identified, and mobility champions were motivated to find solutions, allowing cohesive incorporation of a broad spectrum of priorities. An interprofessional team model is effective to mobilize hospitalized older adults, potentially reducing adverse hospital outcomes. Successful implementation of such programs is dependent on interprofessional collaboration.

